# Supplementary material for: Wood xerogel for fabrication of high-performance transparent wood
Source: Nat Commun. 2023 May 17;14:2827. doi: 10.1038/s41467-023-38481-x (PMC10192348; doi:10.1038/s41467-023-38481-x)
Supplement: Supplementary file 1 — Supplementary Information [file 41467_2023_38481_MOESM1_ESM.pdf]

Supplementary Information for

Wood Xerogel for Fabrication of High-Performance Transparent Wood

Shennan Wang[1], Lengwan Li[2], Li Zha[1], Salla Koskela[1,2], Lars A. Berglund[2], Qi Zhou[1,2]*

[1] Division of Glycoscience, Department of Chemistry, School of Engineering Sciences in Chemistry, Biotechnology and Health, KTH Royal Institute of Technology, AlbaNova University Centre, Stockholm SE-106 91, Sweden.

[2] Wallenberg Wood Science Center, Department of Fibre and Polymer Technology, KTH Royal Institute of Technology, Stockholm SE-100 44, Sweden.

*Corresponding author, e-mail: qi@kth.se

This Supplementary Information includes:

- Supplementary Notes 1–4
- Supplementary Figures 1–12
- Supplementary Tables 1–4
- Supplementary References 1–22

Supplementary Note 1: Effect of crosslinking with trivalent aluminum ion

Balsa wood with densities of 190, 280, and 350 kg m^{-3} was used to prepare the TO-wood xerogels. The density of the xerogel crosslinked with Al^{3+} were slightly lower than that of the corresponding non-crosslinked xerogel, indicating less collapse of pore structures (Supplementary Table 1). As characterized by N_2 physisorption, both non-crosslinked and Al^{3+}-crosslinked TO-wood xerogels presented type IV physisorption isotherms and H3 hysteresis loops, which suggest a mesoporous structure with the presence of macropores larger than 100 nm (Supplementary Fig. 3). The BET specific surface area S_{BET} of the TO-wood xerogels increased with increasing balsa wood density (Supplementary Table 1). The S_{BET} of Al^{3+}-crosslinked TO-wood xerogels from different starting wood densities were all higher than the corresponding non-crosslinked TO-wood xerogels. The highest S_{BET} of 260 m^2 g^{-1} was obtained when high density balsa with Al^{3+} crosslinking was applied. The mesopore volume (V_{meso}), describing the total volume of pores with diameters in the range of 2–50 nm, in TO-wood xerogel (0.374 cm^3 g^{-1}) was 5.3 times larger than the V_{meso} of D-wood xerogel (0.070 cm^3 g^{-1}), indicating a significant increase in the mesoporosity inside the cell wall.

Tensile test of the wood xerogels prepared from balsa wood with a density of 350 kg m^{-3} was performed at 23 °C and 50% relative humidity on a universal tester Instron 5944 (MA, USA) equipped with a 500 N load cell. Samples with dimensions of 50 mm × 3 mm (longitudinal and radial directions) were stretched at a strain rate of 10% min^{-1} in the longitudinal direction (Supplementary Fig. 2a). The non-crosslinked sample showed tensile strength (σ) of 0.80 ± 0.16 MPa, Young's modulus (E) of 0.11 ± 0.01 GPa, and strain-to-failure (ε) of 1.36 ± 0.12 % along the longitudinal direction (Supplementary Fig. 2b). After crosslinking with Al^{3+} ions, the mechanical properties of TO-wood xerogel were significantly improved, showing σ of 4.81 ± 0.19 MPa, E of 0.32 ± 0.04 GPa, and ε of 3.62 ± 0.96 %.

Supplementary Note 2: Infiltration of acrylic resin in the wood xerogels

When both TO-wood and D-wood xerogels were immersed in ABPE resin monomer containing 1 wt.% photoinitiator, air bubbles were observed around both xerogels, indicating the exchange of air in the porous structure with the resin monomers in the surrounding. After 12 hours, TO-wood immersed in resin monomer was almost invisible due to the complete infiltration of the resin while D-wood remained opaque (Supplementary Fig. 4). In addition, contact angle test of ABPE resin droplets (3 μl) on the surfaces of TO-wood and D-wood xerogels was performed (Supplementary Fig. 5). The infiltration of ABPE resin monomer into xerogel surface was much

faster for the TO-wood as the droplet was already absorbed into TO-wood xerogel after 5 s. By contrast, the droplet remained on the D-wood xerogel surface with a contact angle of 58.4° after 10 min.

Supplementary Note 3: Collection of hexane

To verify the collection of hexane by silica gels, the weight change of silica gels before and after the drying of the wood xerogel was measured. The result showed that 94% of the added 100 ml hexane was adsorbed to silica gel, measured by a weight increase of 61.8 g, which is equivalent to 94.35 ml hexane taking the density of hexane as 0.655 g cm^{-3} (Supplementary Fig. 12).

Supplementary Note 4: Measurements of porosity and wood volume fraction in transparent wood samples

The porosities Φ of the wood xerogel/ABPE composites were calculated according to the following equations:

$$\Phi = (1 - (V_{\mathrm{xerogel}} + V_{\mathrm{resin}})/V_{\mathrm{composite}}) \times 100\% \quad (1)$$

$$V_{\mathrm{xerogel}} = W_{\mathrm{xerogel}}/\rho_{\mathrm{xerogel}} \quad (2)$$

$$V_{\mathrm{resin}} = W_{\mathrm{resin}}/\rho_{\mathrm{resin}} \quad (3)$$

$$W_{\mathrm{resin}} = W_{\mathrm{composite}} - W_{\mathrm{xerogel}} \quad (4)$$

Where:

ρ_{xerogel} is the true density of xerogel in equivalent to wood cell wall density (1.5 g cm^{-3}).

ρ_{resin} is the density of neat ABPE resin (1.2 g cm^{-3}).

$V_{\mathrm{composite}}$ is the apparent volume of composite.

V_{xerogel}, and V_{resin} are the calculated volume of xerogel and resin in the composite.

W_{resin}, W_{xerogel}, and $W_{\mathrm{composite}}$ are the experimental weights of resin, xerogel and composite.

The wood volume fraction (V_{f}) in the composite was calculated according to the following equation:

$$V_{\mathrm{f}} = (V_{\mathrm{xerogel}}/V_{\mathrm{composite}}) \times 100\% \quad (5)$$

Supplementary Figures

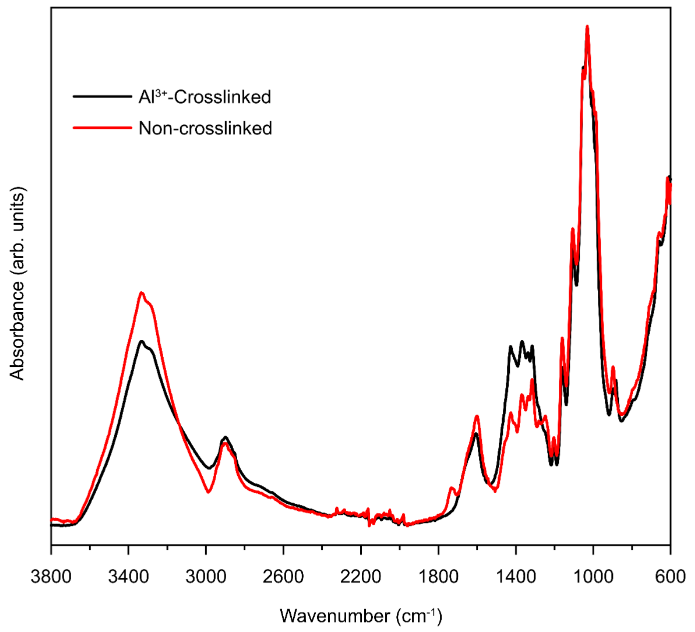

Supplementary Fig. 1 FTIR analysis. Fourier transform infrared spectroscopy (FTIR) spectra of the Al^{3+}-crosslinked and non-crosslinked TO-wood xerogels.

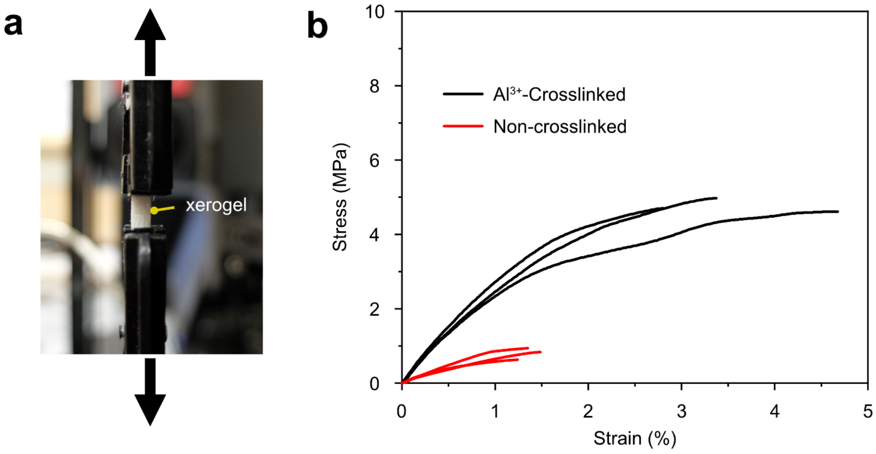

Supplementary Fig. 2 Tensile test for TO-wood xerogels. a Photograph of the tensile test setup with a specimen. **b** Tensile stress-strain curves of the Al^{3+}-crosslinked and non-crosslinked TO-wood xerogels. The starting balsa wood density was 350 kg m^{-3}.

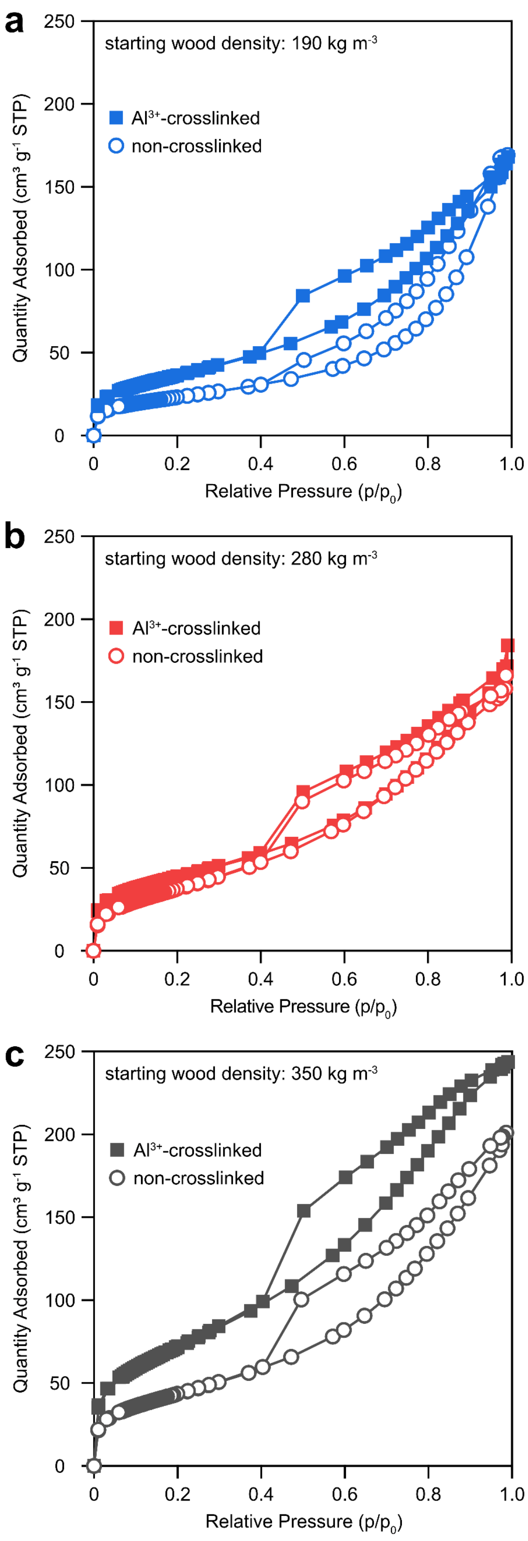

Supplementary Fig. 3 N_2 physisorption. Nitrogen adsorption-desorption isotherms of TO-wood xerogels prepared from balsa wood with starting wood density of (a) 190 kg m^{-3}, (b) 280 kg m^{-3}, and (c) 350 kg m^{-3}. The solid squares represent Al^{3+}-crosslinked TO-wood xerogels and the empty circles represent non-crosslinked TO-wood xerogels.

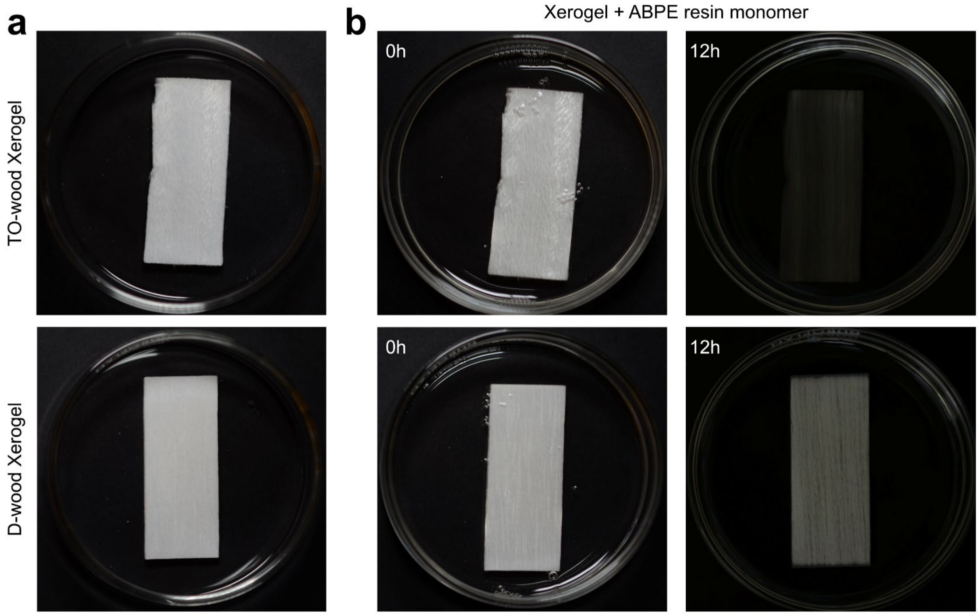

Supplementary Fig. 4 Infiltration of acrylic resin in the wood xerogels. **a** Photographs of the dry TO-wood and D-wood xerogels in glass Pertri dishes. **b** Photographs of the xerogels when the ABPE resin monomers were added and after impregnation for 12 h.

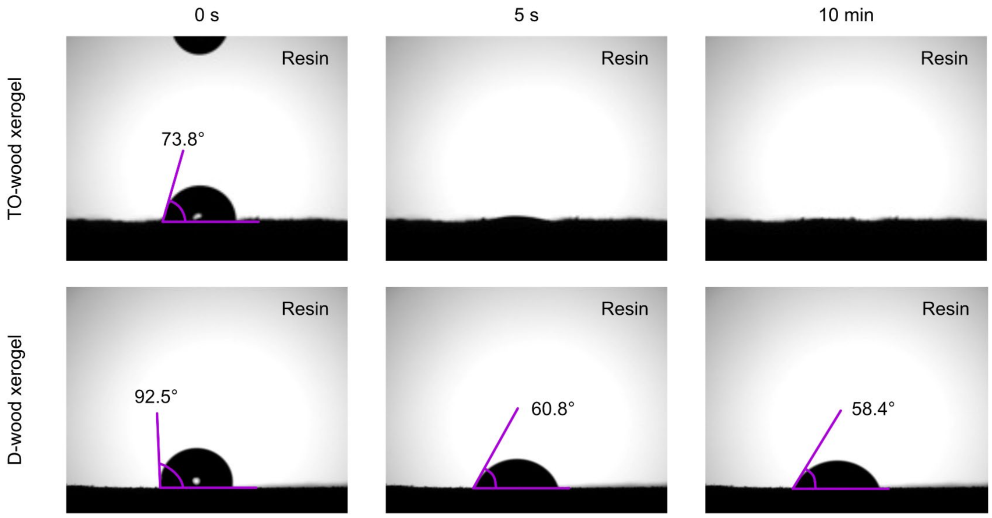

Supplementary Fig. 5 Sessile drop measurements. ABPE resin contact angle images of the TO-wood and D-wood xerogels as a function of time.

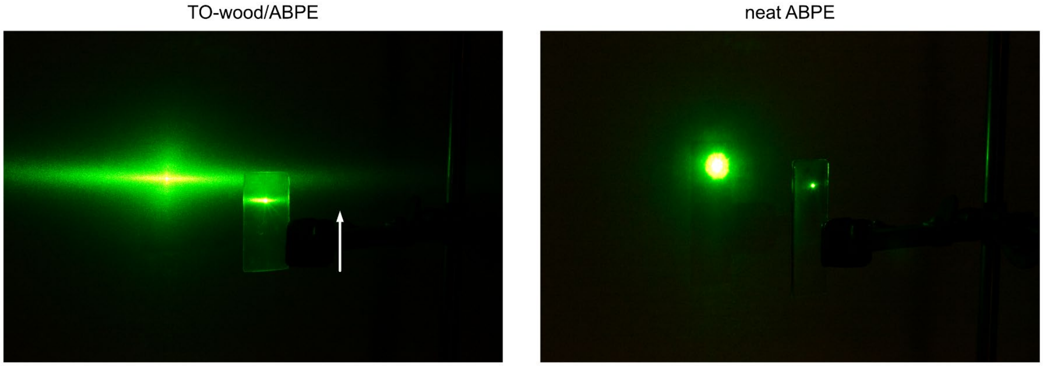

Supplementary Fig. 6 Light scattering effect. Laser light scattering effect of the transparent TO-wood/ABPE composite (wood volume fraction: 12%, thickness: 1.10 mm) and the neat ABPE acrylic resin sheet (thickness: 1.10 mm). Note that the diameter of the laser point is 1 mm, the distance between the sample and the wall is 20 cm. The sample size is 20 × 50 mm^2 and the white arrow indicates the fiber direction.

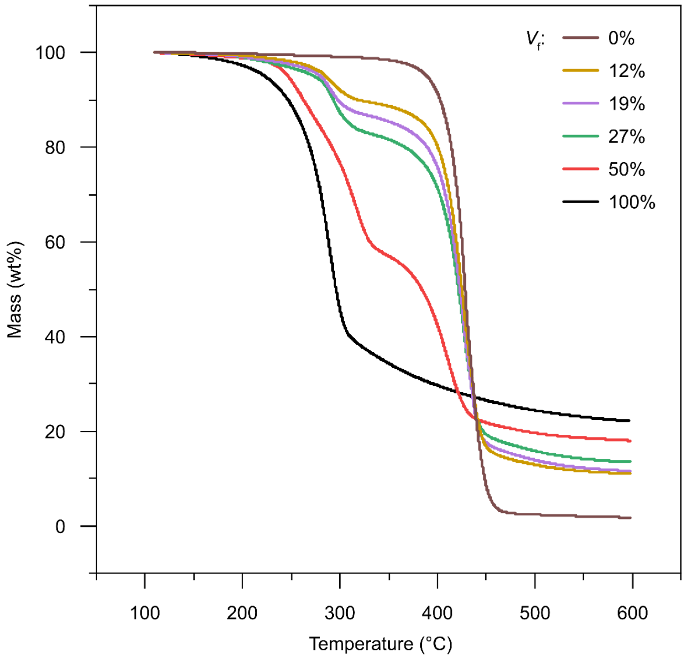

Supplementary Fig. 7 Thermogravimetric analysis (TGA). TGA curves of the neat ABPE resin, the neat TO-wood xerogel, and the TO-wood/ABPE composites with different wood volume fractions (V_f) under nitrogen flow.

Samples	Magnification x50	Magnification x5000
TO-wood/ABPE V_f = 12%	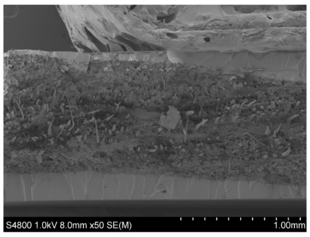	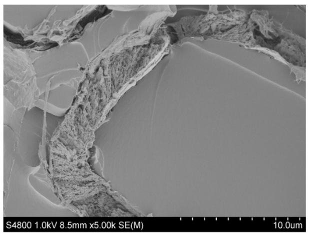
TO-wood/ABPE V_f = 19%	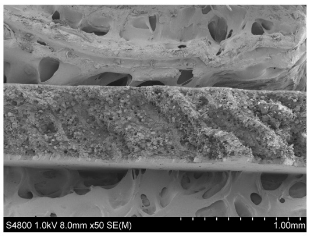	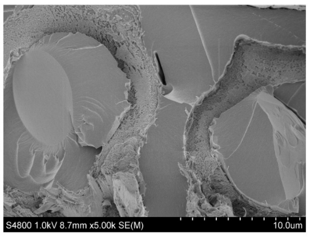
TO-wood/ABPE V_f = 27%	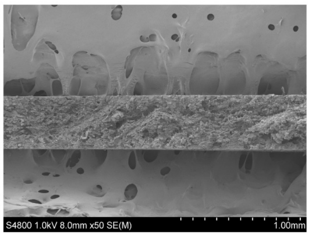	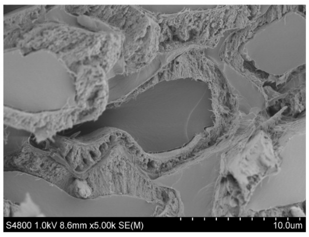
TO-wood/ABPE V_f = 50%	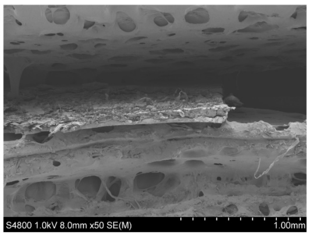	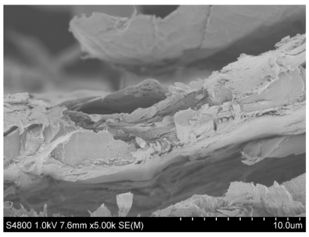
D-wood/ABPE V_f = 17%	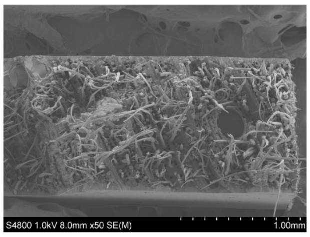	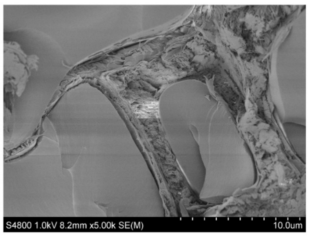

Supplementary Fig. 8 Morphology of tensile fractured surfaces. FE-SEM micrographs of tensile fracture surfaces of transparent TO-wood/ABPE composites with different wood volume fractions (V_f) and the D-wood/ABPE composite.

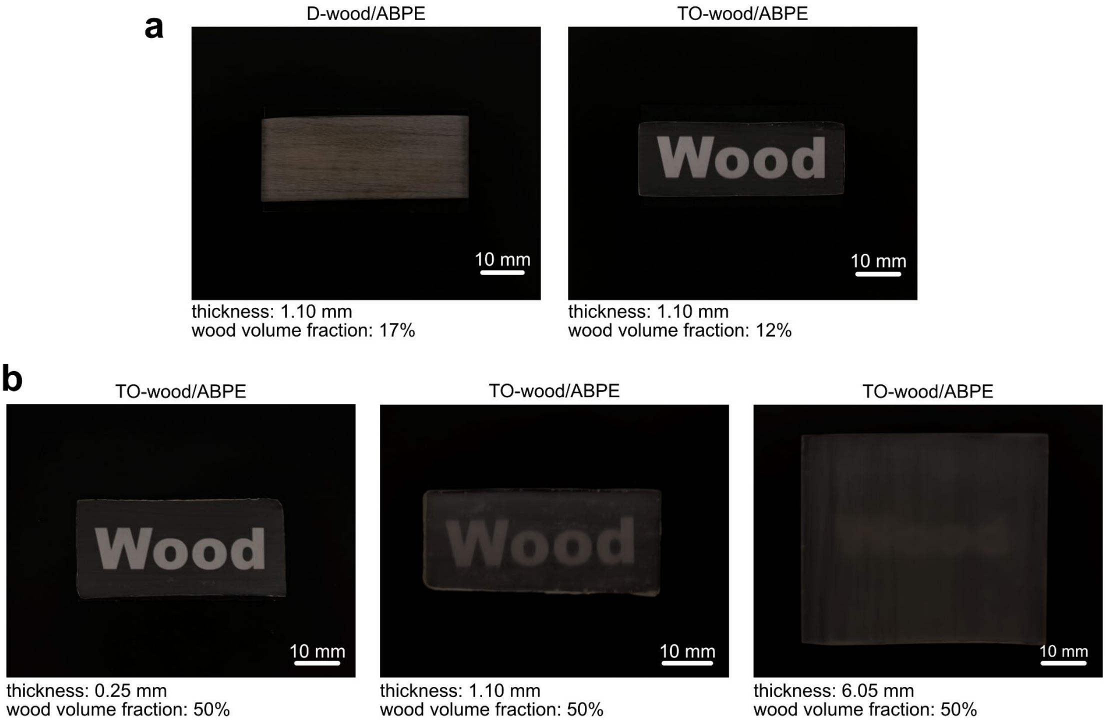

Supplementary Fig. 9 Effect of thickness on optical transparency. a Photographs of the control D-wood/ABPE composite and the transparent TO-wood/ABPE composites shown in Fig. 1a in the main text on black background. **b** Photographs of the TO-wood/ABPE composites with 50% wood volume fraction and different thicknesses of 0.25 mm, 1.10 mm, and 6.05 mm on black background.

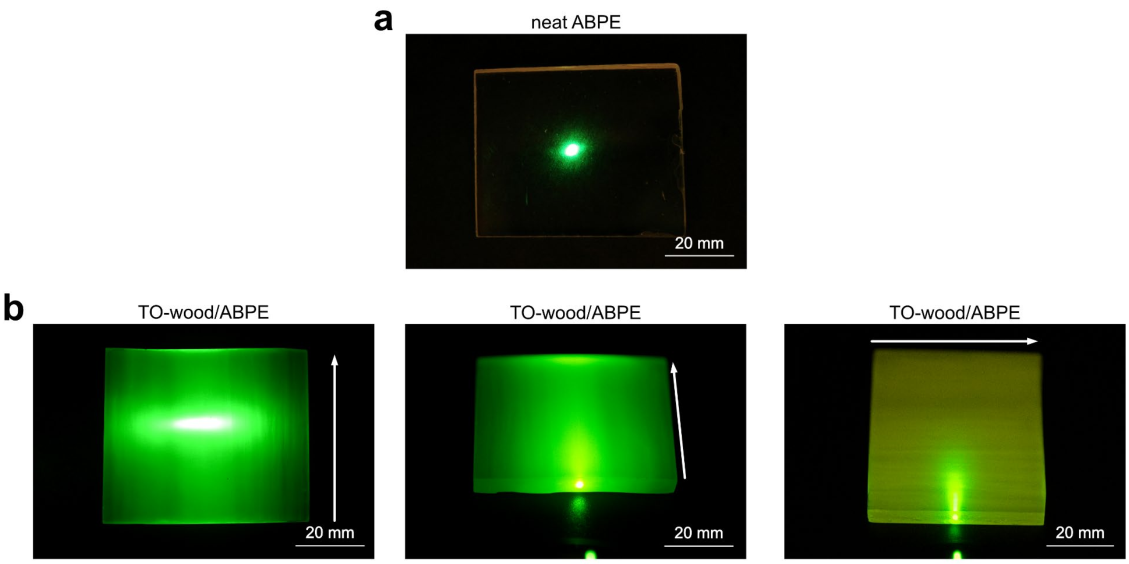

Supplementary Fig. 10 Anisotropic light scattering effect. a Photograph of an incident green laser spotlight pointing to the neat ABPE acrylic resin sheet with a thickness of 6.05 mm. **b** Anisotropic light scattering effect of the transparent TO-wood/ABPE composite sample with a thickness of 6.05 mm and 50% wood volume fraction shown in Fig. 1b in the main text. The diameter of the laser point is 1 mm. The white arrows indicate the longitudinal direction of the fiber cells in transparent wood.

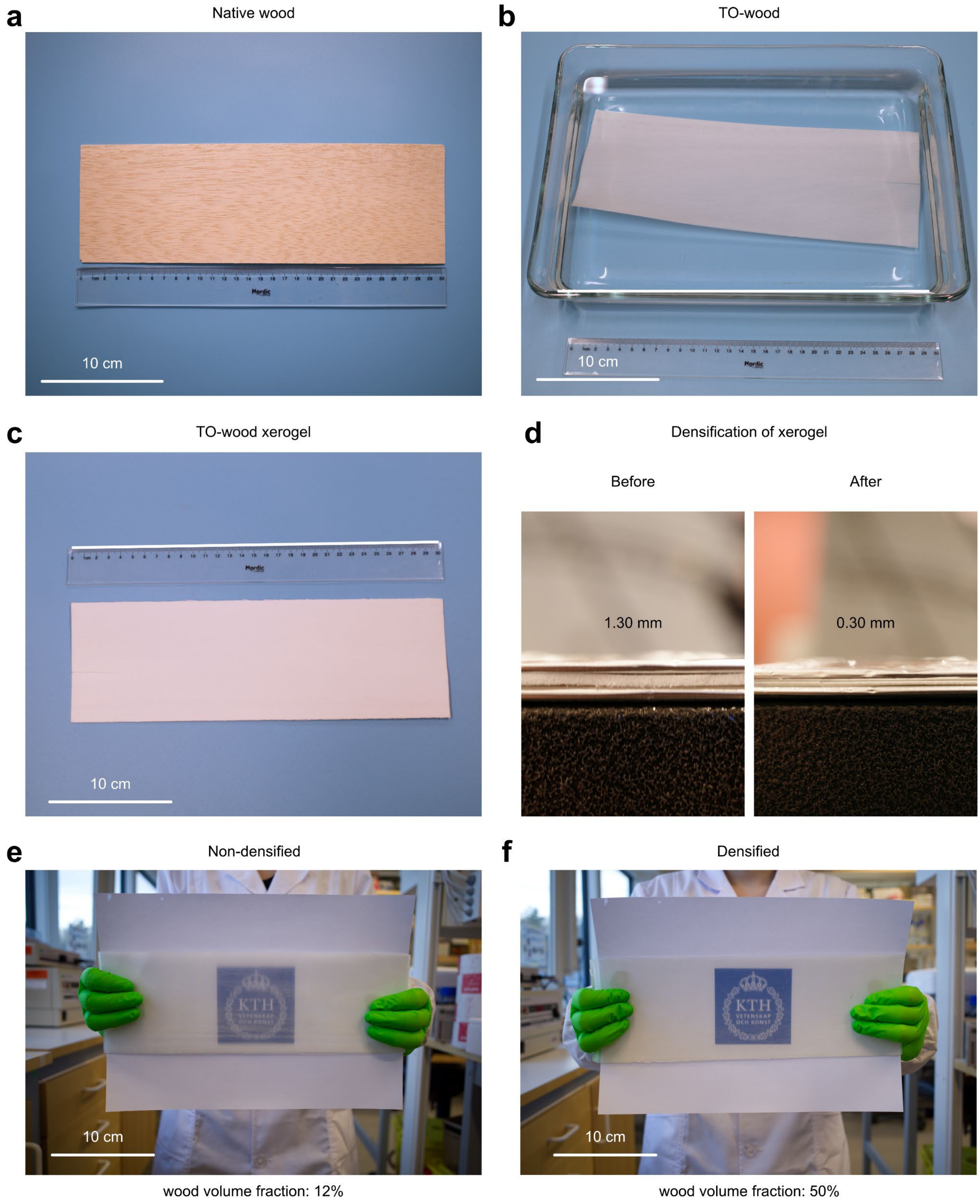

Supplementary Fig. 11 Preparation of large size transparent wood. a Photograph of the native balsa wood board with a dimension of 300 mm × 100 mm × 1.30 mm. **b** Photograph of the TEMPO-oxidized balsa wood board stored in water. **c** Photograph of the TO-wood xerogel prepared from the TEMPO-oxidized balsa wood board. **d** Photographs showing before and after densification of the TO-wood xerogel in thickness direction between two aluminum plates covered with tin foil. **e** Photograph of the transparent wood from the non-densified TO-wood xerogel. **f** Photograph of the transparent wood from the densified TO-wood xerogel.

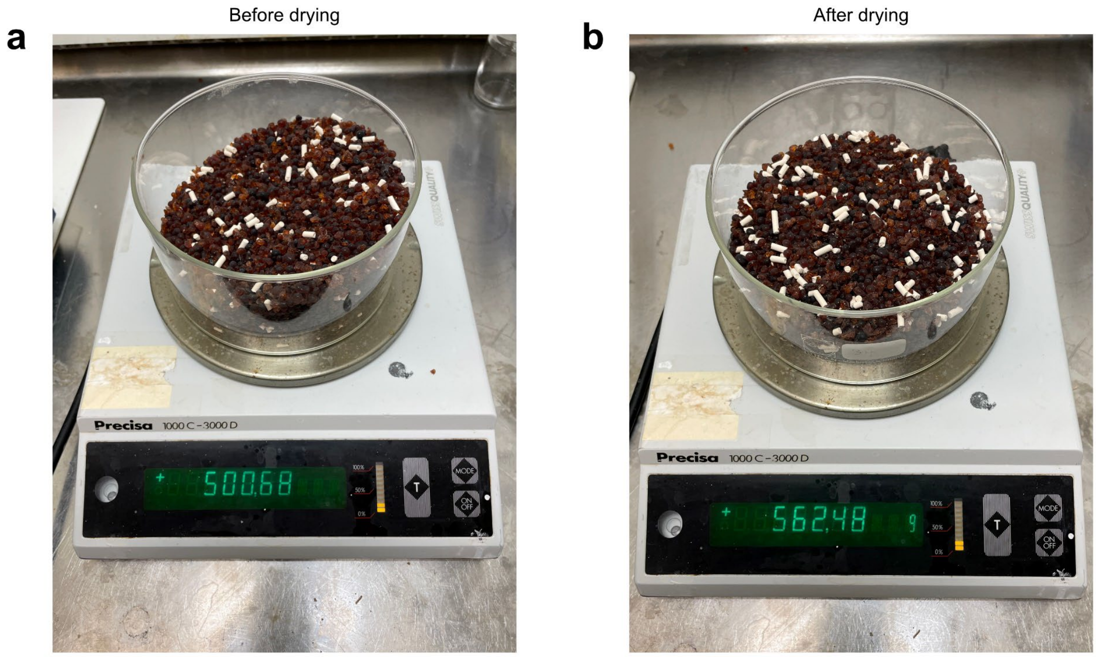

Supplementary Fig. 12 Collection of hexane. a Photograph showing the weight of the silica gel before drying of the wood xerogel with hexane. **b** Photograph showing the weight of the silica gel with absorbed hexane after the wood xerogel was dried.

Supplementary Tables

Supplementary Table 1. Density of the native wood ($\rho_{native\ wood}$), and density ($\rho_{xerogel}$), porosity, BET specific surface area (S_{BET}), and mesopore volume (V_{meso}) of the D-wood xerogel and TO-wood xerogels with and without crosslinking with Al^{3+}.

	$\rho_{native\ wood}$ (kg m^{-3})	Al^{3+}	$\rho_{xerogel}$ (kg m^{-3})	Porosity (%)	S_{BET} (m^2 g^{-1})	V_{meso} (cm^3 g^{-1})
D-wood	350	-	239	84.0	37	0.070
TO-wood	190	No	139	90.7	83	0.244
		Yes	133	91.1	130	0.248
	280	No	168	88.8	148	0.248
		Yes	156	89.6	155	0.249
	350	No	192	87.2	157	0.301
		Yes	185	87.6	260	0.374

Supplementary Table 2. Thickness, density (ρ), wood volume fraction (V_f), porosity (Φ), total transmittance at 550 nm (T_{550nm}), and haze of the TO-wood/ABPE and D-wood/ABPE composites, and neat ABPE acrylic resin sheet.

Samples	Thickness (mm)	ρ (g cm^{-3})	V_f (%)	Φ (%)	T_{550nm} (%)	Haze (%)
TO-wood/ABPE	1.10	1.24	12	0.6	81.1	58.5
	0.70	1.26	19	1.2	83.5	52.7
	0.49	1.28	27	1.0	82.5	46.3
	0.25	1.35	50	1.4	82.1	40.0
	1.10	1.35	50	1.1	72.3	73.9
D-wood/ABPE	1.10	1.25	17	6.3	38.7	95.5
Neat ABPE	1.10	1.20	0	N/A	90.1	1.9

N/A: not applicable.

Supplementary Table 3. Tensile strength (σ), Young's modulus (E), and strain-to-failure (ε) of the TO-wood/ABPE and D-wood/ABPE composites with different wood volume fractions (V_f) along the longitudinal direction (fiber direction), and neat ABPE acrylic resin sheet. (Numbers in parentheses are standard deviations)

Samples	V_f (%)	σ (MPa)	E (GPa)	ε (%)
TO-wood /ABPE	50	259 (14)	29.0 (1.7)	1.4 (0.2)
	27	124 (6)	15.6 (0.4)	0.9 (0.1)
	19	91 (5)	10.5 (0.5)	0.8 (0.1)
	12	59 (3)	7.7 (0.2)	0.8 (0.1)
D-wood /ABPE	17	88 (9)	8.0 (0.5)	1.1 (0.1)
Neat ABPE	0	2 (0)	32.8 (1.9) MPa	5.4 (1.2)

Supplementary Table 4. Mechanical properties including tensile strength (σ) and Young's modulus (E), and optical properties including total transmittance at 550 nm (T_{550nm}) and haze of transparent wood with different polymer matrices, wood volume fractions (V_f), and thicknesses in literatures.

Wood template	Polymer matrix	V_f	σ (MPa)	E (GPa)	T_{550nm}** (%)	Haze (%)	Thickness (mm)	Ref
TO-wood xerogel	ABPE	50%	259	29.0	82.1	40.0	0.25	This work
TO-wood xerogel	ABPE	50%	259	29.0	72.3	73.9	1.10	This work
D-wood	PMMA	25%	262.7	19.3	70	70	0.65	1
D-wood	PMMA	N/A	45.92	2.66	80.6	N/A	0.5	2
D-wood	PMMA	19%	90.1	3.59	85.0	71.0	1.2	3
D-wood	PMMA	12%	62.5	4.3	90-95	50-60	0.8	4
D-wood	PMMA	N/A	60.1	2.67	86	90	5	5
D-wood	PEG/PMMA	25.8%	70.5	14.9	68	77	1.5	6
D-wood	PMMA	6.4%	41.4	4.8	70.6	76.3	2	7
D-wood	PLIMA	26%	146.6	12.6	87	46	1.2	8
D-wood	Epoxy	N/A	45.38	2.37	90	~100	3	9
D-wood	Epoxy	N/A	76.28	1.4	68.2	N/A	2.5	10
D-wood	Epoxy	2.5%	43.39	N/A	90	10	0.7	11
D-wood	Thiol-ene	N/A	61	3.6	66	47	1.1	12
D-wood	Thiol-ene	28%*	179	12.3	86	50	1.1	13
D-wood	Thiol-ene	6.8%	50.7	4.11	~85	N/A	1	14
D-wood	Thiol-ene	4.3%	59	3.4	90	36	1.2	15
D-wood	PVA	29%	39.9	1.51	80	90	1	16
D-wood	PVA	N/A	143	3.85	91	15	0.8	17
D-wood	Polyimide	N/A	169	2.11	$75_{750\ nm}$	75	0.2	18
D-wood	MF	25%	60	11.1	74	66	1.2	19
esterified D-wood	PLIMA	26%	173.6	17.3	90	30	1.2	8
partially delignified wood	PMMA	N/A	171.4	N/A	$61_{800\ nm}$	N/A	0.42	20
partially delignified wood	Epoxy	N/A	91.95	N/A	$80_{600\ nm}$	93	2	21
lignin modified wood	Epoxy	30%	46	N/A	90	60	1	22

*Only weight fraction data available; ** Value is obtained at 550 nm if not specified.
PMMA: polymethyl methacrylate, PVA: polyvinyl alcohol, PLIMA: poly(limonene acrylate), PEG: polyethylene glycol, MF: melamine formaldehyde.

Supplementary References

1. Jungstedt, E., Montanari, C., Östlund, S. & Berglund, L. Mechanical properties of transparent high strength biocomposites from delignified wood veneer. *Compos. Part A Appl. Sci.* **133,** 105853 (2020).
2. Gan, W. et al. Luminescent and transparent wood composites fabricated by poly(methyl methacrylate) and γ-Fe_2O_3@YVO_4:Eu^{3+} nanoparticle impregnation. *ACS Sustainable Chem. Eng.* **5,** 3855–3862 (2017).
3. Li, Y., Fu, Q., Yu, S., Yan, M. & Berglund, L. Optically transparent wood from a nanoporous cellulosic template: Combining functional and structural performance. *Biomacromolecules*. **17,** 1358–1364 (2016).
4. Fu, Q. et al. Transparent plywood as a load-bearing and luminescent biocomposite. *Compos. Sci. Technol.* **164,** 296–303 (2018).
5. Yu, Z. et al. Transparent wood containing CsxWO3 nanoparticles for heat-shielding window applications. *J. Mater. Chem. A*. **5,** 6019–6024 (2017).
6. Montanari, C., Li, Y., Chen, H., Yan, M. & Berglund, L. A. Transparent wood for thermal energy storage and reversible optical transmittance. *ACS Appl. Mater. Interfaces*. **11,** 20465–20472 (2019).
7. Montanari, C., Olsén, P. & Berglund, L. A. Interface tailoring by a versatile functionalization platform for nanostructured wood biocomposites. *Green Chem.* **22,** 8012–8023 (2020).
8. Montanari, C., Ogawa, Y., Olsén, P. & Berglund, L. A. High performance, fully bio-based, and optically transparent wood biocomposites. *Adv. Sci.* **8,** 2100559 (2021).
9. Zhu, M. et al. Highly anisotropic, highly transparent wood composites. *Adv. Mater.* **28,** 5181–5187 (2016).
10. Zhang, L. et al. Transparent wood composites fabricated by impregnation of epoxy resin and W-doped VO_2 nanoparticles for application in energy-saving windows. *ACS Appl. Mater. Interfaces*. **12,** 34777–34783 (2020).
11. Jia, C. et al. Clear wood toward high-performance building materials. *ACS Nano*. **13,** 9993–10001 (2019).
12. Samanta, A. et al. Reversible dual-stimuli-responsive chromic transparent wood biocomposites for smart window applications. *ACS Appl. Mater. Interfaces*. **13,** 3270–3277 (2021).
13. Samanta, A. et al. Charge regulated diffusion of silica nanoparticles into wood for flame retardant transparent wood. *Adv. Sustainable Syst.* **6,** 2100354 (2022).

14. Hoglund, M. et al. Facile processing of transparent wood nanocomposites with structural color from plasmonic nanoparticles. *Chem. Mater.* **33,** 3736–3745 (2021).
15. Hoglund, M., Johansson, M., Sychugov, I. & Berglund, L. A. Transparent wood biocomposites by fast UV-curing for reduced light-scattering through wood/thiol-ene interface design. *ACS Appl. Mater. Interfaces*. **12,** 46914–46922 (2020).
16. Subba Rao, A. N., Nagarajappa, G. B., Nair, S., Chathoth, A. M. & Pandey, K. K. Flexible transparent wood prepared from poplar veneer and polyvinyl alcohol. *Compos. Sci. Technol.* **182,** 107719 (2019).
17. Mi, R. et al. A clear, strong, and thermally insulated transparent wood for energy efficient windows. *Adv. Funct. Mater.* **30,** 1907511 (2019).
18. Chen, L. et al. A flame-retardant and transparent wood/polyimide composite with excellent mechanical strength. *Compos. Commun.* **20,** 100355 (2020).
19. Samanta, P. et al. Fire-retardant and transparent wood biocomposite based on commercial thermoset. *Compos. Part A Appl. Sci.* **156,** 106863 (2022).
20. Wu, J. et al. Impact of delignification on morphological, optical and mechanical properties of transparent wood. *Compos. Part A Appl. Sci.* **117,** 324–331 (2019).
21. Mi, R. et al. Scalable aesthetic transparent wood for energy efficient buildings. *Nat. Commun.* **11,** 3836 (2020).
22. Xia, Q. et al. Solar-assisted fabrication of large-scale, patternable transparent wood. *Sci. Adv.* **7,** eabd7342 (2021).
